# The effect of titanium dioxide nanoparticles on mice midbrain substantia nigra

**DOI:** 10.22038/ijbms.2019.33611.8018

**Published:** 2019-07

**Authors:** Zahra Heidari, Abbas Mohammadipour, Parisa Haeri, Alireza Ebrahimzadeh-bideskan

**Affiliations:** 1 Department of Anatomy and Cell Biology, School of Medicine, Mashhad University of Medical Sciences, Mashhad, Iran; 2 Microanatomy Research Center, School of Medicine, Mashhad University of Medical Sciences, Mashhad, Iran

**Keywords:** Dark neurons, Mice, Substantia nigra, Titanium dioxide-nanoparticles, Tyrosine hydroxylase-neurons

## Abstract

**Objective(s):**

Widely used Titanium dioxide nanoparticles (TiO_2_) enter into the body and cause various organ damages. Therefore, we aimed to study the effect of TiO_2_ on the substantia nigra of midbrain.

**Materials and Methods:**

40 male BALB/c mice were randomly divided into five groups: three groups received TiO_2_ at doses of 10, 25, and 50 mg/kg, the fourth group received normal saline for 45 days by gavage, and control group (without intervention). Then, Motor tests including pole and hanging tests were done to investigate motor disorders. The animal brain was removed for histological purposes. Accordingly, immunohistochemistry was performed to detect tyrosine hydroxylase positive cells, and then toluidine blue staining was done to identify dark neurons in the substantia nigra. Eventually, the total number of these neurons were counted using stereological methods in different groups.

**Results:**

The results showed that the time recorded for mice to turn completely downward on the pole in the TiO_2_-50 group increased and also the time recorded for animals to hang on the wire in the hanging test significantly decreased (*P<*0.05) in comparison with other groups. Also, the average number of tyrosine hydroxylase positive neurons in TiO_2_-25 and TiO_2_-50 groups significantly decreased as compared to the TiO_2_-10 and control groups (*P<*0.05). The total number of dark neurons in the TiO_2_-25 and TiO_2_-50 groups was substantially higher than the TiO_2_-10, control and normal saline groups (*P<*0.05).

**Conclusion:**

Our findings indicated that TiO_2_, depending on dose, can cause the destruction of dopaminergic neurons and consequently increase the risk of Parkinson’s disease.

## Introduction

Nanotechnology is a collection of techniques and methods for producing nano-sized materials with new usages. Nowadays there are studies on various nanoparticles, many of which are regarding Titanium dioxide (TiO_2_) nanoparticles (1-3). TiO_2_ nanoparticles have photocatalytic and photoelectric effects, and they are ultra-violet agents (4), and because of their specific physical and chemical properties, these nanoparticles are widely used to manufacture cosmetic and hygienic products, sunscreens, toothpastes, and equipment used in the medical field (5-7). The small size and smaller diameter of the nanoparticles allow them to easily enter the body at different stages of manufacture, transportation, and consumption (8). These nanoparticles enter the central nervous system (CNS) by passing through the blood-brain barrier (BBB) and during inhalation by the olfactory nerve (9). It has been proven that by entering the brain, these nanoparticles accumulate in the brain and lead to brain weight loss, inflammation, immunological response, cell injury, and impairment in brain function (5, 10). Exposure to these nanoparticles during pregnancy and lactation can reduce the proliferation of hippocampal cells and impair memory and learning in the offspring (11, 12). Shimizu *et al*. (2009) showed that administration of TiO_2_ leads to changes in the expression of genes involved in brain development, cell death, and response to oxidative stress in the prenatal period (13). These nanoparticles also induce oxidative stress in glial cells by decreasing the antioxidant enzymes gene expression, lipid peroxidation, and mitochondrial depolarization (14). *In vitro* studies show that TiO_2_ nanoparticles lead to neuronal damage by stimulating microglia to produce ROS (15). These nanoparticles also can damage the neurons through DNA methylation (16). Also, previous studies confirmed that administration of TiO_2 _nanoparticles in animals has led to changes in trace elements in the brain and even reduces memory and learning in them (17). 

The substantia nigra (SN) is a midbrain large motor nucleus that is composed of two main components: the pars compacta (dense part) and pars reticularis. The dens part contains dopaminergic (DA) neurons that produce neuromelanin, which protects these neurons against oxidative-related damages by inactivation of free radicals (18). Besides, the dense DA neurons of the dense part send the fibers to the striatum, which release dopamine in the neural terminals. Destruction of pars compacta (SNpc) DA neurons is followed by a reduction of dopamine synthesis and release in the striatum and finally, leads to the appearance of Parkinson’s disease (PD) symptoms (19). 

PD is known as a common motor syndrome of the CNS that is diagnosed with symptoms such as bradykinesia, rigidity, instability in condition, and tremor (20). Recent studies have shown that in addition to genetic causes, environmental factors by damaging the DA neurons can also be risk factors for PD (21).

 With regards to the advancements and developments of nanotechnology and the widespread usage of TiO_2_ nanoparticles, as well as the lack of sufficient investigations on the effects of TiO_2_ nanoparticles on the SN of the midbrain, the present research was designed to study the neurotoxic effects of these nanoparticles on DA neurons. For this purpose, pole and hanging tests were used to investigate motor disorders. Furthermore, the immunohistochemistry and toluidine blue staining were used to determine the number of tyrosine hydroxylase (TH) positive neurons and dark neurons, respectively. 

## Materials and Methods

***Chemical and preparation***

TiO_2_ nanoparticles used in this study were characterized with a size of 10 nm, purity of 99%, specific surface area>150 m^2^/g (Brunauer-Emmett-Teller (BET) equation), and anatase crystalline phase obtained from the Nano Lima Corporation (Iran). Primary antibody (anti TH) was purchased from Abcam, and secondary antibody (goat anti-rabbit IGg) was purchased from Dako.

 These nanoparticles were prepared immediately before being used as a form of suspension in the normal saline and administrated as a form of gavage.

***Animals***

Forty male Balb/c mice aged 7–8 weeks and weighing 25–30 gr were obtained from the animal center of Mashhad University of Medical Science, Mashhad, Iran. During the study, animals were kept in the standard conditions in terms of temperature (20 ± 2 °C) and 12 hr light/dark cycle, and *ad libitum* access to water and food.

After adaptation, animals were randomly divided into five equal groups (n=8):

1) TiO_2_-10 group: received 10 mg/kg of TiO_2_ nanoparticles by gavage for 45 days (17, 22).

2) TiO_2_-25 group: received 25 mg/kg of TiO_2_ nanoparticles by gavage for 45 days. 

3) TiO_2_-50 group: received 50 mg/kg of TiO_2_ nanoparticles by gavage for 45 days (17).

4) Normal saline group: received 0.5 ml normal saline by gavage for 45 days.

5) Control group: without any intervention.

***Motor tests***

At the end of experiments, motor tests were performed to determine the motor disorders for each of the five groups.

*Pole test*

Mice were placed on top of a pole 55 cm high and 8 mm in diameter. The time spent by all animals to turn downward was recorded entirely. Finally, the average time was compared between the five studied groups. Then the average time spent in each group was compared to the other groups (23).

*Hanging test*

In this test, animals were placed on a 100 cm wire, 40 cm high, and 2 mm in diameter. Then the hanging wire was rotated to place the animals in a hanging condition. The time that each mice was able to keep itself hanging was carefully recorded. Finally, the average time of each group was compared to the other groups

***Tissue preparation***

After motor tests, the animals were anesthetized using chloroform, and their skull was cut along the sagittal suture, and the animal brain for histological studies was removed, fixed in 10% formalin solution, and then the tissues were dehydrated, cleared, and embedded in alcohol, xylene, and paraffin, respectively. 5 mm cuts were serially prepared from the samples. A total of 8 sections with an interval of 10 sections were selected randomly and used for immunohistochemistry to detect TH positive (TH^+^) neurons and toluidine blue staining to identify dark neurons in the SN of the midbrain.

***Immunohistochemistry***

For immunostaining, sections were deparaffinized with xylene and then rehydrated through descending concentrations of alcohol. Then the sections were washed with Phosphate buffered saline (PBS) for 5 min and antigen retrieval was done at 96/4 ^°^C for 15 min and followed by incubation with Bovine Serum Albumin (BSA) (0.01 g BSA+ 1000 µl PBS+ 10 µl Triton x) at room temperature (20-25 ^°^C) for 30 min. In the next step, the slides were placed in hydrogen peroxide (H_2_O_2_, 0.03% solution) in PBS for 15 min to block endogenous peroxidase. After that, incubation with goat serum was made at room temperature for 20 min, and then the samples were incubated with primary antibody (anti-TH, 1:70, ab191486, Abcam Company) overnight. In the next step, the slides were washed with PBS and exposed to goat anti-rabbit IGg secondary antibody (ab97051, Abcam Company) with 1:100 concentrations for 90 min. All samples were treated with 3, 3’-Diaminobenzidine (DAB) and H_2_O_2_ at room temperature for 10 min. After washing the samples with running water, they were counterstained with Haematoxylin, dehydrated in increasing graded alcohols, cleared in xylene solution, and mounted with cover glasses. Eventually, the sections were prepared to study by optical microscopy (24, 25).

***Toluidine blue staining***

For toluidine blue staining, at first, sections were cleared with xylene, rehydrated with descending alcohols and then stained with 1% toluidine blue solution for 20 sec. Afterward, the tissue slides were washed with distilled water (DW), dehydrated in increasing graded alcohols, cleared in xylene, and mounted with cover glasses. Then, they were prepared for study by optical microscopy (26, 27).

***Measurements***

First, with the aid of a light microscope (Olympus BX51, Japan) equipped with a camera attached to a computer, the colored samples of SNpc were photographed with ×20 and ×40 lenses. Images were transferred to the computer monitor. Then, TH^+^ cells and dark neurons were counted by using the counting frame. The number of TH^+^ cells and dark neurons were separately calculated for each sample by using the following formula:

$N_{A}=\frac{\sum \bar{Q}}{a/\mathrm{f.}\sum P}$

In this formula, “NA” represents the number of neurons in area, “∑Q ®” refers to the sum of counted particles in certain sections, “a/f” is related to the area associated with each frame, and “∑P” means the sum of frame-associated points hitting the defined space (28).

***Statistical analysis***

All data obtained from experiments were analyzed using SPSS 16 software and one-way ANOVA and Tukey’s tests. *P*<0.05 was considered significant difference between groups. 

## Results

***Assessment of motor skill tests***

*Evaluation of pole test*

**Figure 1:**
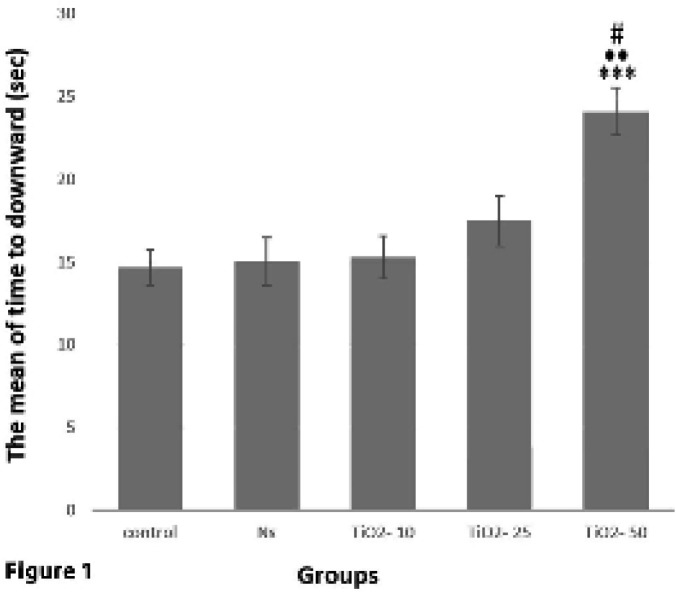
Comparison of the average time spent by animals to turn downward from the pole in the pole motor test in different studied groups. The mean time taken in the TiO_2_-50 group shows a significant rise compared with TiO_2_-10 (••*P<*0.01), TiO_2_-25 (#*P<*0.05), control, and normal saline (***P<0.001) groups

Analysis of pole test data indicated a significant rise in meantime recorded for mice to turn downward in the TiO_2_-50 group compared with TiO_2_-10 (*P*<0.01), TiO_2_-25 (*P*<0.05), control (*P*<0.001) and normal saline (*P*<0.001) groups. While no significant difference was found between the other groups (Figure 1).

*Evaluation of hanging test*

**Figure 2:**
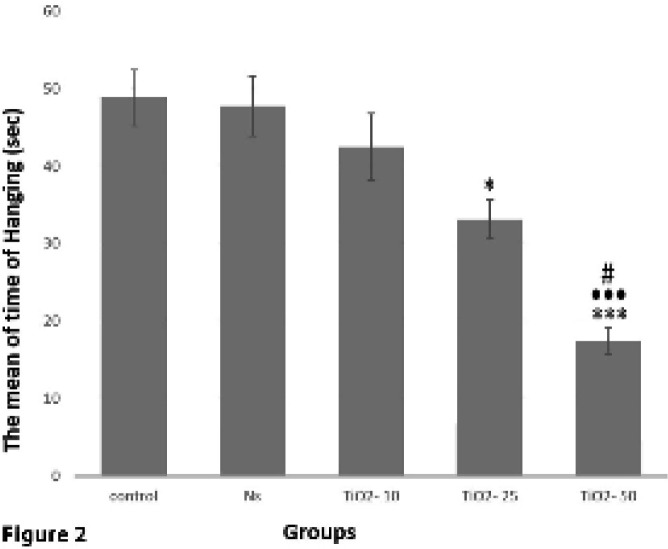
Comparison of the average time recorded for animals in the hanging test in different groups. The mean time taken to hang on a wire in TiO_2_-50 group showed a significant decrease as compared to TiO_2_-10 (•••*P<*0.001), TiO2-25 (#*P<*0.05), control, and normal saline groups (***P<0.001). In TiO_2_-25 group the time to stay suspended was significantly lower than those of control and normal saline groups (^*^P<0.05)

The results obtained from hanging test analysis demonstrated a significant reduction of mean time taken for mice to hang in the TiO_2_-50 group in comparison with control and normal saline groups (*P*<0.001). Also, the mean time taken to hang in the TiO_2_-50 group revealed a significant decrease in comparison with TiO_2_-10 (*P*<0.001) and TiO_2_-25 groups (*P*<0.05). This time in TiO_2_-25 group showed a significant decrease in comparison with control and normal saline groups (*P*<0.05) (Figure 2).

***Histopathological study***

*Immunohistochemical evaluation*

**Figure 3:**
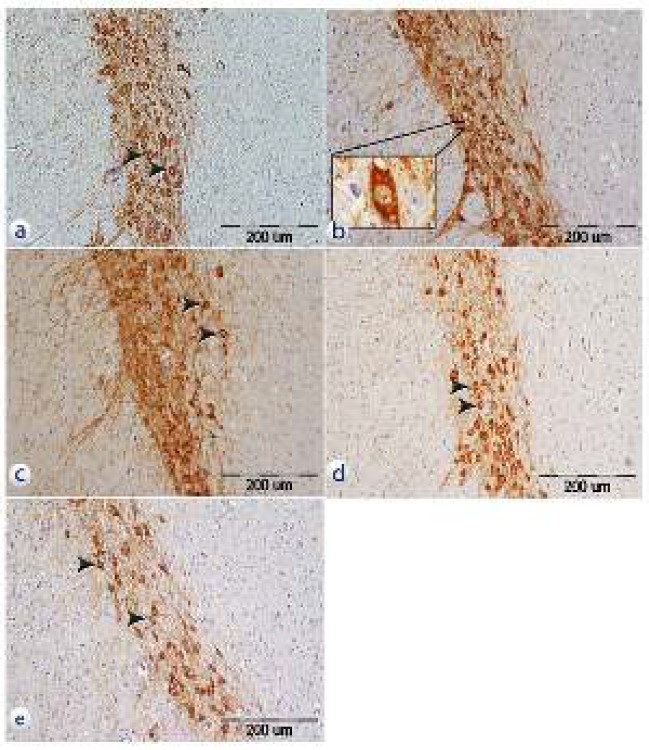
Photomicrograph of the tissue sections from SNpc that have been incubated with anti-TH antibody in different groups. a: control group, b: normal saline group, c: TiO_2_-10 group, d: TiO_2_-25 group, e: TiO_2_-50. 20X, Olympus BX51, Japan, Scale bar= 200 µm. Arrows show TH+ neurons

Assessment of tissue sections using the immunohistochemistry technique revealed that TH^+^ neurons of the SNpc were visible in studied groups. TH^+^ neurons were less visible in samples belonging to TiO_2_-50 and TiO_2_-25 groups in comparison with control and normal saline groups. While, TH^+^ neurons were more visible in TiO_2_-10, control, and normal saline groups (Figure 3).

**Figure 4.**
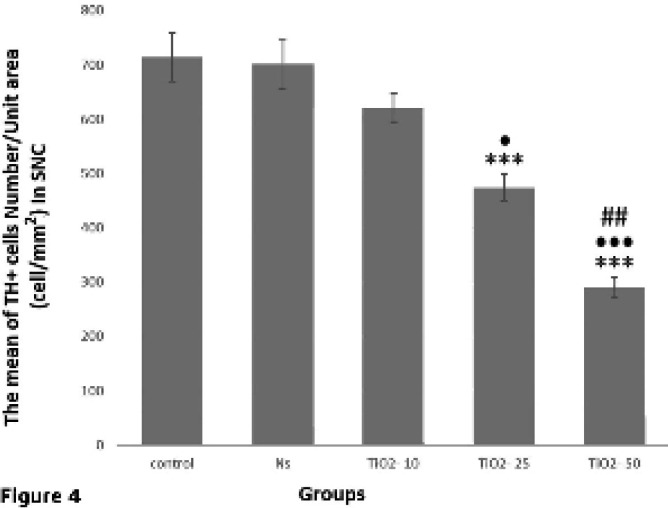
Assessment of the average number of SNpc TH+ neurons in different groups. The average number of TH+ neurons in the TiO_2_-50 group exhibited a significant decrease compared to control and normal saline groups (***P<0.001). Also, the mean number of SNpc TH+ neurons in the TiO_2_-50 group was significantly reduced as compared to both TiO_2_-10 (•••*P<*0.001) and TiO_2_-25 (## *P<*0.01) groups. In addition, the average number of TH+ cells in the TiO_2_-25 group had a significant decrease compared with control (***P<0.001), normal saline (***P<0.001), and TiO_2_-10 groups (•*P<*0.05)

The results of TH^+^ neuron count in the immunohistochemistry method showed that the number of TH^+^ neurons in the SNpc of TiO_2_-25 and TiO_2_-50 groups was significantly lower than those in the control and normal saline groups (*P*<0.001). Besides, there was no significant difference in the TiO_2_-10 group in comparison with control and normal saline groups; while there was a significant decrease in the number of TH^+^ neurons in TiO_2_-50 group compared to TiO_2_-10 (*P*<0.001) and TiO_2_-25 (*P*<0.01) groups. Also, the number of these neurons in the TiO_2_-25 group was significantly lower than TiO_2_-10 group (*P*<0.05) (Figure 4).

*Toluidine blue staining*

**Figure 5:**
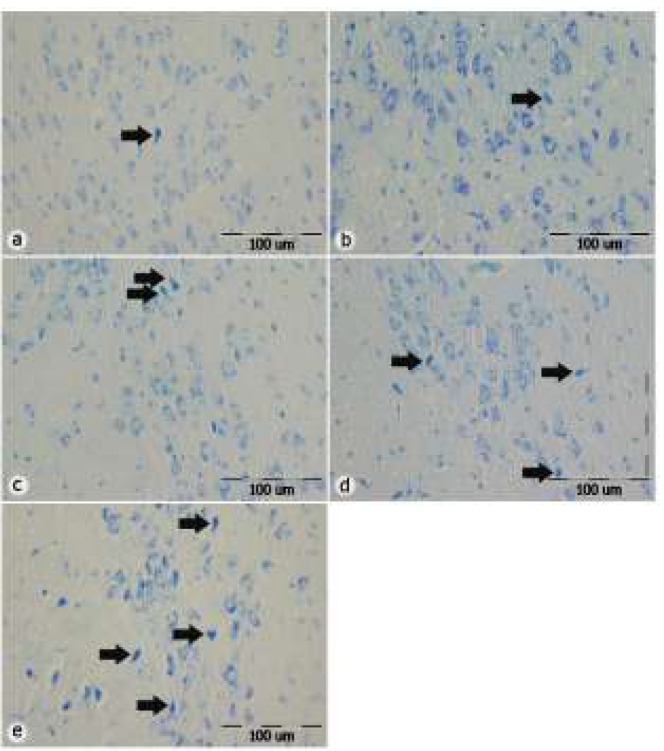
Photomicrograph of toluidine blue staining of tissue sections from SNpc in different groups. The photos illustrate an increase in the number of dark neurons in the groups which received TiO_2_ in comparison with control and normal saline groups. a: control group, b: normal saline group, c: TiO_2_-10 group, d: TiO_2_-25 group, e: TiO_2_-50. Scale bar=100 µm, 20X, Olympus BX51, Japan, Arrows show dark neurons

Examination of brain tissue slides using toluidine blue staining displayed that dark neurons in the SNpc are evident in different groups. However, the number of dark neurons in samples from TiO_2_-50 and TiO_2_-25 groups was more than this number in other TiO_2_-10, control, and normal saline groups (*P*<0.001) (Figure 5).

**Figure 6:**
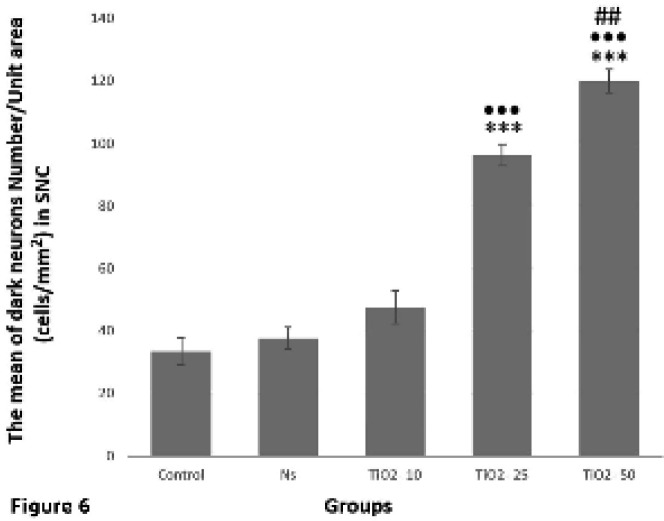
Evaluation of the average number of dark neurons in the SNpc in studied groups. Accordingly, the mean number of dark neurons in the TiO_2_-25 and TiO_2_-50 groups significantly increased as compared to the control (***P<0.001), normal saline (***P<0.001), and TiO_2_-10 groups (•••*P<*0.001). Also, there was a significant increase in the mean number of dark neurons in TiO_2_-50 group in comparison with TiO_2_-25 group (##*P<*0.01)

Also, the results demonstrated that the number of dark neurons in TiO_2_-25 and TiO_2_-50 groups significantly increased as compared to TiO_2_-10 group (*P*<0.001). In the TiO_2_-50 group, the number of these neurons was significantly higher than that of the TiO_2_-25 group (*P* = 0.003) (Figure 6).

## Discussion

The purpose of this study was to investigate the effect of TiO_2_ nanoparticles on the DA neurons and also formation of dark neurons in the SNpc. Our results showed that the administration of TiO_2_ nanoparticles with different doses could reduce the number of TH^+^ neurons and increase the number of dark neurons. Previous *in vitro* studies have also shown that TiO_2_ nanoparticles increase oxidative stress, impair mitochondrial function, resulting in reduced survival of PC12 cells (the cells similar to DA neurons) in the growth medium (29). Our results showed that there was no significant difference in the number of TH^+^ neurons in the group receiving 10 mg/kg titanium as compared to the control and normal saline groups. However, in TiO_2_-25 and TiO_2_-50 groups, there was a significant decrease in the number of these neurons in comparison with control and normal saline groups, which can indicate the potential for damage of TiO_2_ nanoparticles is somewhat dose-dependent.

 These nanoparticles, after entering the body, by reducing the expression of the proteins involved in the tight junction, lead to a change in the integrity of BBB and through it enter the brain (30). After passing through the BBB, the nanoparticles enter the cells due to their tiny size. Following the accumulation of TiO_2_ nanoparticles in the brain, the content of some electrolytes such as calcium is changed, and brain damage caused by calcification in neurocytes and the proliferation of ependymal cells directly or indirectly leads to disturbed homeostasis of trace elements, enzymes, and neurotransmitters including glutamate, acetylcholine, and dopamine (17).

 DA neurons of the midbrain are responsible for the synthesis of dopamine and TH (a key enzyme for dopamine production) and also are the primary source of dopamine in the CNS (31). Although these neurons are only about 1% of the total neurons in the brain, they play a critical role in the normal function of the motor loop of the brain, and also they control many activities of the brain, including voluntary movements (32). The motor loop of the brain plays an essential role in the controlling of movements, and its damage can lead to motor disorders like PD. This loop has two direct and indirect paths. On the direct path, the fibers are sent from the cortex to the striatum, and from there, sent into the internal segment of Globus Pallidus (Gpi). Then Gpi sends fibers to the thalamus and thalamus sends the fibers to the motor cortex. In normal conditions, this pathway increases the input to the cerebral cortex that leads to an increase in the transmission of commands from the cortex to the muscles for voluntary movement during activity. The result of the indirect movement of the motor loop is the reduction of cortical input and failure in sending commands from the cortex at rest time. The DA neuron axons of SNpc release about 80% of the total dopamine of the brain in the striatum (33, 34). Reducing the release of dopamine leads to the elimination of the working balance of these two pathways by increasing the activity of the indirect pathway and reducing the activity of the direct pathway, which results in the loss of control on the basal nucleus and inability to perform effective movements, which cause the appearance of PD symptoms (23, 35).

 In PD, voluntary movements are slowed down because of damage to the direct pathway of the motor loop and also due to damage to the indirect pathway, limbs tremor occurs at the rest time. Environmental factors play a significant role in the development of CNS diseases and nanoparticles can be one of the important issues affecting the CNS due to their specific characteristics (small size and large surface) and also worldwide usage (32, 36).

 In the present study, it was also found that TiO_2_ nanoparticles reduce the number of TH^+^ neurons in the SN. Several possibilities exist regarding the mechanisms of neuronal damage of these nanoparticles: for example, it has been confirmed that administration of TiO_2_ nanoparticles in mice causes a collapse in the brain electrolytes balance by increasing the levels of calcium and sodium and decreasing the amount of magnesium, potassium, and zinc. This collapse is also due to disruption of the operation of sodium-potassium, potassium-magnesium, and calcium pumps (17). Because of the importance of proper functioning of these pumps and the balance of electrolytes in the neurons, the collapse in their balance leads to neuronal damage. It has been proven that in PD, the activity of the sodium-potassium pump is impaired and this point is essential (37). Another important mechanism through which TiO_2_ does neuronal damage is the formation of neuroinflammation. These nanoparticles increase the level of interleukin 6 and also NF-kB in the brain and result in activation of inflammatory reactions (38). On the other hand, it has been reported that one of the most important factors in the process of developing PD is the development of inflammatory reactions in the DA neurons (39). Another substantial mechanism for TiO_2_ neurotoxic effects is to increase the oxidative stress factors and reduce the antioxidant factors of the cells. By increasing the amount of O_2_^-^, H_2_O_2_, and MDA, these nanoparticles lead to cellular damage (40). Additionally, it has been disclosed that DA neurons of midbrain are highly vulnerable to oxidative stress and in other words, oxidative stress is one of the leading causes for the development of PD (41). In addition to the mechanisms mentioned above, increased accumulation of α-Synuclein in neurons following the administration of TiO_2_ nanoparticles is another important factor. The results of the Wu and Xiu studies in 2016 showed that in the *in vitro* environment, TiO_2_ increased the accumulation of α-Synuclein in PC12 cells (42). It has been confirmed that increase and accumulation of α-Synuclein is one of the most important events in PD. The excessive increase in α-Synuclein prevents the release of dopamine and reduces the amount of dopamine in the brain. It also leads to degeneration of DA neurons over time (43). Therefore, as it is known, there is a large relationship between the neurotoxic mechanisms of TiO_2_ nanoparticles and the mechanisms of PD appearance.

 The results acquired from behavioral pole test of the present study showed that the time taken until the animal falls from the pole in the TiO_2_-50 group significantly increased. In addition, the results of the hanging test indicated a significant decrease in the time until the animal stays on the wire in the TiO_2_-50 group. Previous studies have also shown that the mice exposed to high doses of TiO_2_ show signs of brain damage such as tremor and lethargy (44); considering that PD detection is possible when about 50% of DA neurons are degraded (45). The outcomes of our study also showed that in the TiO_2_-50 group, the number of DA neurons reduced and as a result, the amount of dopamine intensively decreased and PD symptoms were observed. 

An essential component for examining pathological changes in the CNS is the dark neuron. The structure and function of the dark neurons will change after the damage, and they will be wrinkled, basophilic and dark blue (46, 47). The result of our study showed that following the administration of titanium, the number of dark neurons in the SNpc significantly increased. Previous studies also indicate that TiO_2_ nanoparticles result in the death of neurons by increasing the activity of apoptosis enzymes such as Caspase-3 and Caspase-9, increasing Bax, and decreasing Bcl_2_ (48, 49).

## Conclusion

According to our investigation, it can be concluded that TiO_2_ nanoparticles have the potential function for damaging the DA neurons of SNpc. Excessive access of these nanoparticles to DA neurons may also increase the risk of PD.

## Conflicts of interest

No conflicts of interest.
